# Epitopes identified in GAPDH from *Clostridium difficile* recognized as common antigens with potential autoimmunizing properties

**DOI:** 10.1038/s41598-018-32193-9

**Published:** 2018-09-17

**Authors:** Agnieszka Razim, Katarzyna Pacyga, Małgorzata Aptekorz, Gayane Martirosian, Andrzej Szuba, Edyta Pawlak-Adamska, Monika Brzychczy-Włoch, Andrzej Myc, Andrzej Gamian, Sabina Górska

**Affiliations:** 1 Hirszfeld Institute of Immunology and Experimental Therapy, Polish Academy of Sciences, Department of Immunology of Infectious Diseases, Laboratory of Medical Microbiology, Wroclaw, Poland; 2 Department of Medical Microbiology, School of Medicine in Katowice, Medical University of Silesia, Katowice, Poland; 3 Division of Angiology, Wroclaw Medical University, Wroclaw, Poland; 4 Department of Internal Medicine, 4th Military Hospital in Wroclaw, Wroclaw, Poland; 5 Hirszfeld Institute of Immunology and Experimental Therapy of the Polish Academy of Sciences, Department of Experimental Therapy, Laboratory of Immunopathology, Wroclaw, Poland; 6 Department of Molecular Medical Microbiology, Chair of Microbiology, Jagiellonian University Medical College, Krakow, Poland; 7 Research Associate Scientist Emeritus, University of Michigan, Nanotechnology Institute for Medicine and Biological Sciences, Ann Arbor, MI 48109 USA

## Abstract

*Clostridium difficile* (CD) infections are a growing threat due to the strain resistance to antibiotic treatment and the emergence of hypervirulent strains. One solution to this problem is the search for new vaccine antigens, preferably surface-localized that will be recognized by antibodies at an early stage of colonization. The purpose of the study was to assess the usefulness of novel immunoreactive surface proteins (epitopes) as potential vaccine antigens. Such approach might be tough to pursue since pathogens have acquired strategies to subvert adaptive immune response to produce humoral response against non-essential proteins for their survival. In this study CD surface proteins were isolated, immunoreactive proteins identified and mapped to select potential epitopes. The results of the study exclude the use of CD glyceraldehyde 3-phosphate dehydrogenase as a vaccine antigen, especially as a whole protein. Sequences P9 (^201^AAGNIVPNTTGAAKAI^218^) and P10 (^224^KGKLDGAAQRVPVVTG^241^) recognized by patients sera are conserved and widespread among CD strains. They show cross-reactivity with sera of people suffering from other bacterial infections and are recognized by sera of autoimmune disease patients. Our study documents that special care in analyzing the sequence of new epitope should be taken to avoid side effects prior to consider it as a vaccine antigen.

## Introduction

Designing new microbiological vaccines is a complicated and risky process due to the fact that microorganisms use devious techniques of avoiding the immune system. One of the basic mechanisms is changing the expression of antigens present on the surface of the pathogen named as immunological decoy^1^. As an example, *Mycobacterium tuberculosis* (Mtb) expresses Ag85b at a high level at the beginning of infection which leads to T cell response against this protein. When the infection establishes Mtb switches off Ag85b expression and T cell response is no longer a threat^2^. Another strategy is to introduce point mutations in antibody-binding regions^3^. Above tactics cause the use of proteins as a vaccine antigen extremely difficult. One of the proposed solutions is to use conserved and essential proteins.

Glyceraldehyde-3-phosphate dehydrogenase EC 1.2.1.12 (GAPDH) is one of the essential proteins taking part in glycolysis, present in almost all organisms. Recent reports show that this is not the only role and that it possess many other functions like cell signaling, interaction with other proteins, control of gene expression and also takes part in microbial virulence^4^. The protein is no longer considered as solely intracellular, it might be secreted on the cell surface and plays its additional roles as a moonlighting protein. GAPDH is present on the surface of Gram-positive pathogenic strains where it takes part in colonization and manipulation of host cells^5^. It can bind fibronectin, lysozyme, laminin, cytoskeletal proteins^6^, interact with human plasminogen, helps adhesion to pharyngeal cells^7^, also can support escaping host immune system^8^. Identification of so many new functionalities, especially in pathogenic microorganisms, leads to the suggestion that GAPDH might be a suitable vaccine candidate since it is essential for pathogen viability. It was proposed as vaccine antigen against *Schistosoma mansoni*^9^, *Edwardsiella tarda*^10^ and *Streptococcus pneumoniae*^11^. So far, the possibility of using GAPDH as a CD vaccine antigen has not been studied.

There are advantages and disadvantages of GAPDH being such a widespread protein. It was already proposed as a suitable candidate for multivalent vaccine antigen against microbial infection in aquaculture^12^. But, from the other side, it was suggested that because of GAPDH high sequence homology between bacterial strains and human it should be carefully analyzed for possible autoimmunoreactivity^13^ since it could lead to autoimmune disease. GAPDH is already reported as a protein implicated in numerous autoimmune disorders. GAPDH-reactive autoantibodies were found in the Cerebrospinal Fluid (CSF) of patients with multiple sclerosis^14^. Autoantibodies to GAPDH were also reported in CSF of patients with lupus^15^. The mechanism which leads to production of these autoantibodies is still unknown but recently, some authors suggested that these autoantibodies can be primarily initiated by T and B cells reacting to foreign GAPDH, present on the surfaces of parasites, bacteria and fungi^16,17^.

The aim of these studies was to evaluate the effectiveness of immunoreactive surface antigens from *Clostridium difficile* (CD) as potential vaccine antigens. CD is a Gram-positive, strictly anaerobic, spore-forming bacterium that is widely recognized as one of the most common causes of hospital acquired infections. It can cause a wide range of diseases from mild antibiotic-associated diarrhea (AAD) to severe pseudomembranous colitis in immunocompromised patients^18^. The increase of severity and frequency of *Clostridium difficile* infections (CDI) over last few years makes the need for protective and therapeutic vaccines more urgent. In the current research GAPDH was identified as an immunoreactive protein reacting with antibodies from umbilical cord blood sera collected from mothers without the signs of CDI. GAPDH sequence was thoroughly analyzed *in silico* which resulted in a selection of potentially immunoreactive epitopes in the form of 16-mer peptides which were synthesized and mapped using PEPSCAN method and ELISA. Peptides were tested for immunoreactivity using blood sera from CD infected patients resulting in two potential epitopes. Those two epitopes were further investigated to exclude cross-reactivity with other pathogenic and non-pathogenic species, and autoimmunoreactivity.

## Results

### GAPDH is an immunoreactive protein recognized by umbilical cord blood sera

**Figure 1:**
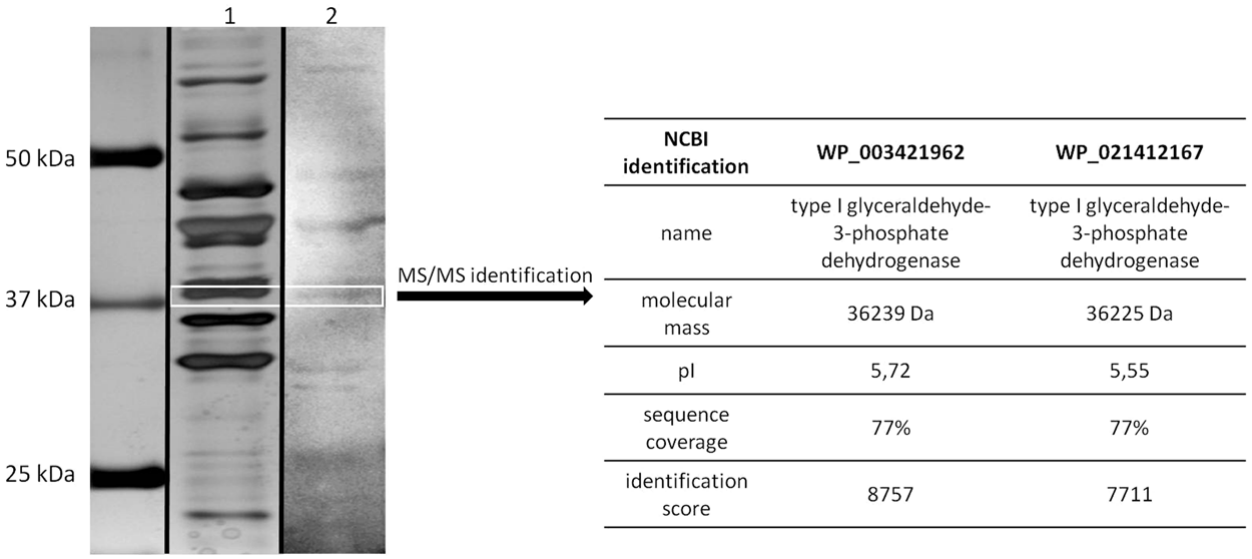
Results of protein isolation and identification. Proteins were extracted using 1 M LiCl, separated in 12,5% gels SDS-PAGE and silver-stained, lane 2. Separated proteins were analyzed by Western blot using umbilical cord blood pooled sera, lane 3. Full-length uncropped gel and blot are included in Supplementary Figs 1 and 2. The white box indicate band selected for MS/MS identification. Results of protein identification are showed in the table on the right.

Proteins isolated from CD with 1 M LiCl were separated using SDS-PAGE and their immunoreactivity was analyzed by Western blotting. The electrophoresis profile is consistent with previous experiments performed with *C. difficile* 630^19^ (Fig. 1) which belongs to the 027 ribotype. However, a novel approach was used to search for new immunoreactive proteins which is the use of umbilical cord blood sera. This method is routinely used in our laboratory^20,21^. It revealed a set of immunoreactive proteins (Fig. 1). A band that corresponds to a mass of 37 kDa was excised and analyzed by LC-MS/MS. Protein was identified by comparison of peptides masses in UniProt database (NCBI) using MASCOT and statistical analysis. One of the identified proteins was assigned as type I glyceraldehyde 3-phosphate dehydrogenase (GAPDH) with a molecular mass of 36.2 kDa, pI 5.72, identified with high coverage of 77% and identification score of 8757 (Fig. 1). The second protein found in the same gel band was also identified as GAPDH Type I but with slightly different pI and lower identification score.

**Figure 2:**
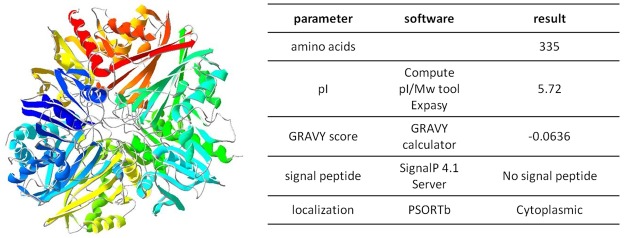
Model of GAPDH and sequence characteristics. Model was prepared based on sequence with 74.55% identity which was *Streptococcus agalactiae* GBS GAPDH (template: 5jyf.1.A).

Sequence analysis between 50 different strains of CD shows that GAPDH occurs as two types and both types are conservative between toxigenic and non-toxigenic CD strains (data not shown). The structure of CD GAPDH is unknown, so that a structural model was built based on a template sequence appointed by BLAST. The structure model of GAPDH was prepared based on sequence with 74.55% identity which is *Streptococcus agalactiae* GAPDH (template: 5jyf.1.A). The protein was modeled as a homotetramer, with no ligands. It is composed in 26% of α-helix, 23% of β-strands and 51% of loops (Fig. 2). Detailed bioinformatics analysis showed that there are not known signal peptides in the protein sequence and that localization is predominantly cellular. This structural characteristics was exploited in planning peptides for epitope mapping.

### *Clostridium difficile* GAPDH as a moonlighting protein

To our knowledge there are no reports about possible alternative role of GAPDH in CD. We performed detailed analysis of GAPDH sequence using available bioinformatics tools and compared obtained results with known physical features of intracellular proteins that moonlight on the cell surface^22^. Most of the moonlighting proteins does not have signal sequence, they comprise of 200–800 amino acids with theoretical pI between 4.5 and 6.5 and the Grand Average of Hydropathy (GRAVY) score of moonlighting proteins is negative or zero. CD GAPDH meets all of the above criteria (Fig. 2). CD GAPDH comprises of 335 amino acids with theoretical pI of 5.72 and GRAVY score of −0.0636. We performed sequence alignment of GAPDH from CD and moonlighting GAPDH from various strains gathered in Supplementary Table S1. The percent identity created by Clustal2.1 ranged from 55.32% for *Bacillus anthracis* GAPDH up to 75.15% for *Streptococcus* species which indicates high sequence similarity between some strains that have moonlighting GAPDH. To sum up, since we have identified GAPDH in the mixture of surface proteins, obtained by a gentle wash-off method^19^ and the protein have similar characteristics of moonlighting proteins and shares with them high sequence similarity we suggest that GAPDH from CD might be one of the moonlighting proteins.

### Epitope mapping using CDI patient sera reveals highly immunoreactive peptide sequences

**Figure 3:**
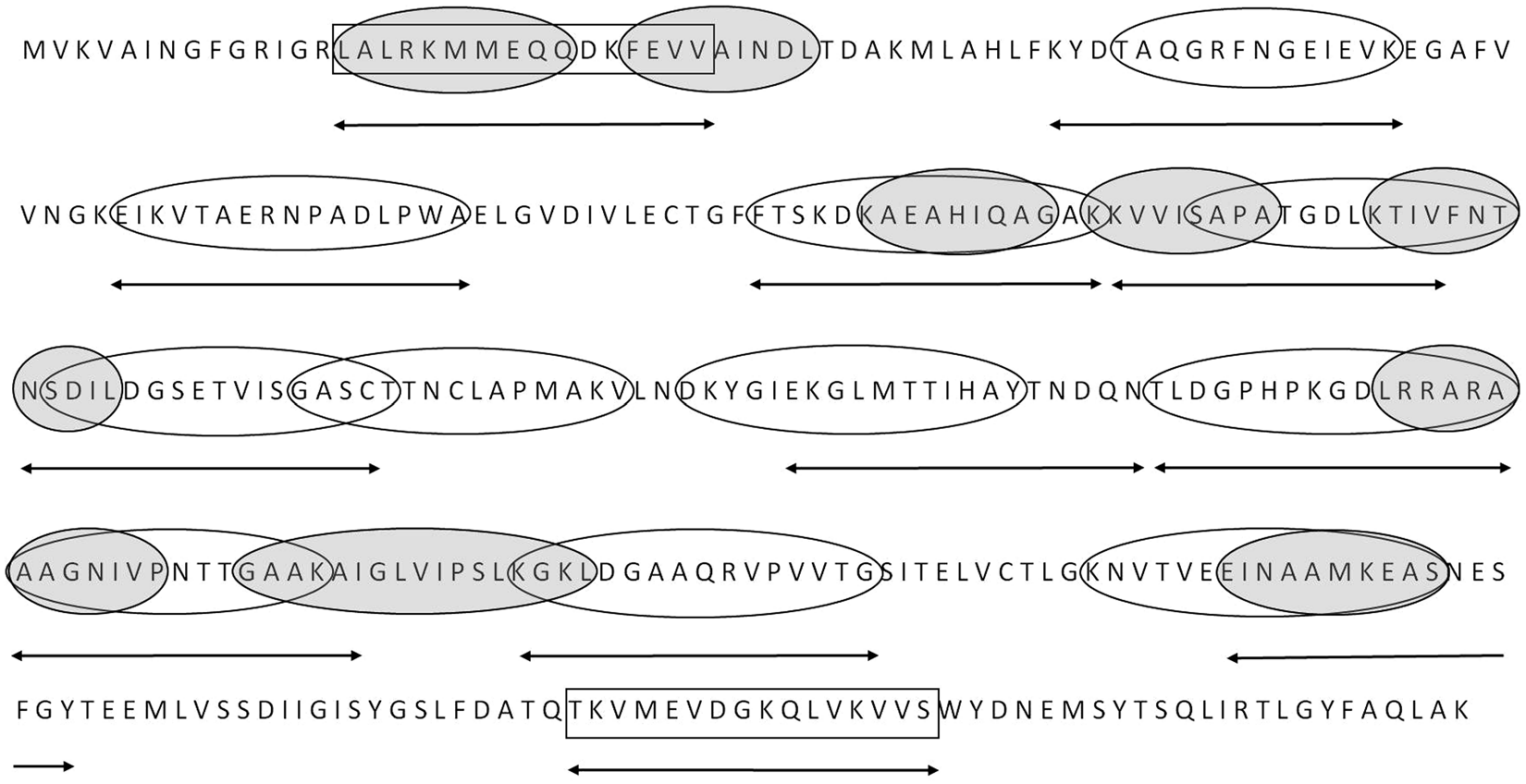
The design of peptides selected for mapping. Figure shows *C. difficile* GAPDH sequence and peptide sequences that were considered for mapping - identified by software as probable epitopes. Empty ovals – B-cell epitopes; shaded ovals – T-cell epitopes; rectangles – sequences in exposed loops. Sequences indicated by arrows are the one selected for chemical synthesis.

**Figure 4:**
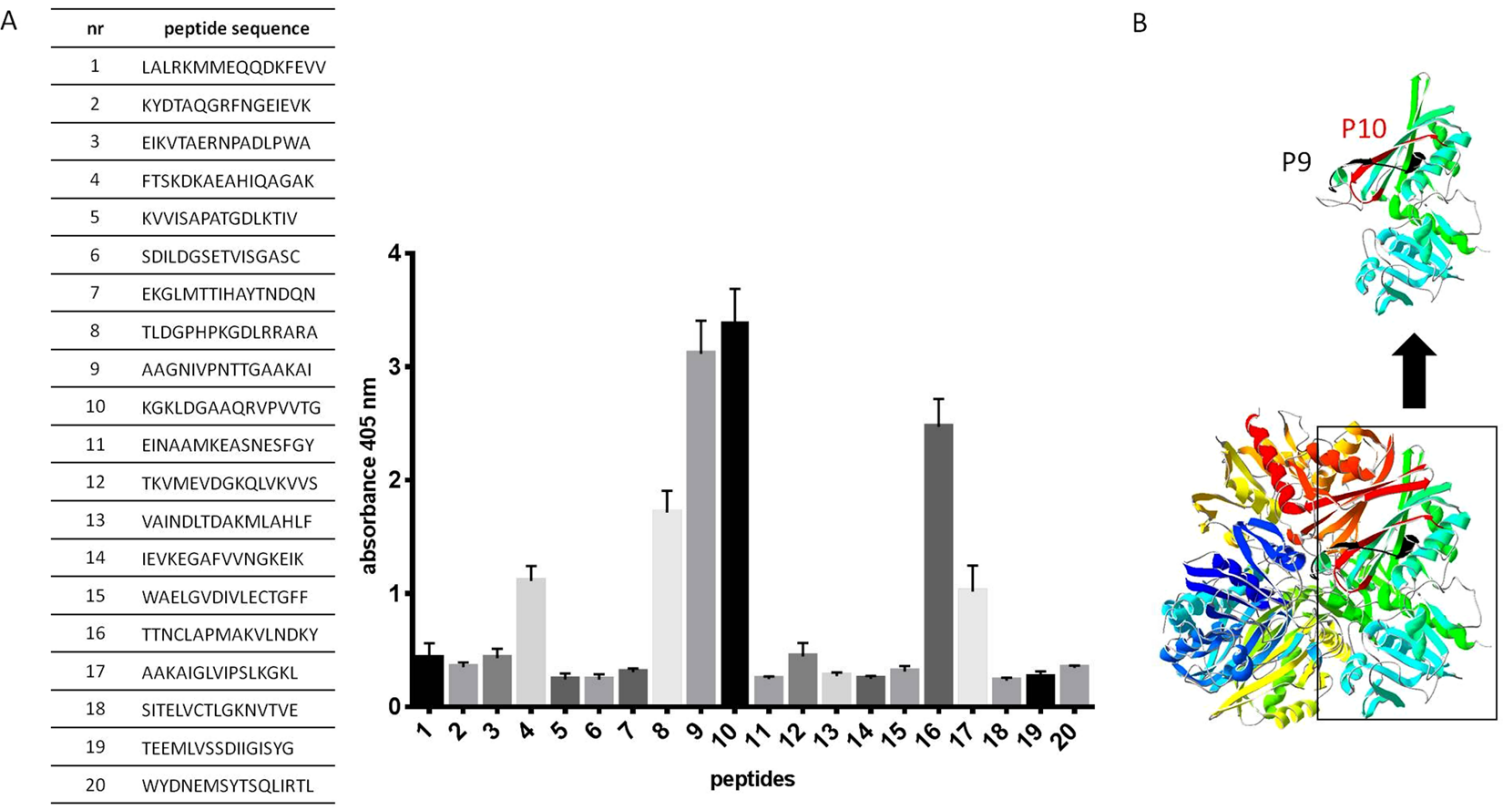
Results of epitope mapping using pooled sera from CDI patients (n = 10). (**A**) shows results of ELISA performed using peptides bound to the pins on 96 well plate. Primary antibody was detected by anti-human IgG-AP conjugated goat antibody which reacts with AP Yellow substrate. The result of colorimetric reaction was measured by reading absorbance in 405 nm. The figure shows results from three independent measurements, means with SD. (**B**) shows the localization of peptides P9 and P10.

Based on structural model of CD GAPDH (Fig. 2) and bioinformatics analysis of B-cell and T-cell linear epitopes we qualified 20 peptides 16-amino acids long for PEPSCAN synthesis and mapping (Fig. 3). Pin-bound peptide ELISA with pooled CDI patient sera showed various peptide activity. Two peptides, which are named as numbers 9 (^201^AAGNIVPNTTGAAKAI^218^, P9) and 10 (^224^KGKLDGAAQRVPVVTG^241^, P10) have the highest immunoreactivity (Fig. 4A), they were 10 to 11 times more active than non-active peptides (peptides of absorbance less than 1). Both epitopes were selected as potential B cell epitopes and their fragments as T cell epitopes during *in silico* analysis. The localization within the protein of peptides P9 and P10 is shown on Fig. 4B. There are also two less active peptides numbers 8 and 16, which activity is 5.5 and 8 time more than non-active peptides. We decided to focus on P9 and P10 in next experiments, also we selected peptide number 5 as a non-active control (^115^KVVISAPATGDLKTIV^132^, P5).

### Peptides 9 and 10 contain common epitopes

**Figure 5:**
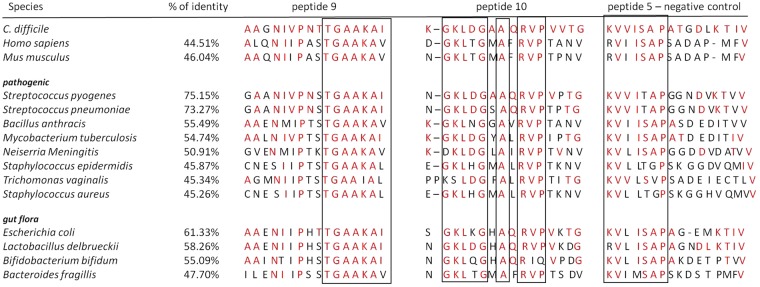
Alignment of *Clostridium difficile* GAPDH peptide sequences and GAPDH from other species. % identity of GAPDH sequences compared to *Clostridum difficile* GAPDH sequence. Amino acids in the red are the one shared with *C. difficile* and selected strains. Sequences in the frames are most conservative between strains. Selected strains include human, mouse, pathogenic strains that express moonlighting GAPDH and most prevalent strains from gut flora.

We compared GAPDH sequences of virulent strains that are known to express moonlighting GAPDH with CD protein (Fig. 5). The percent of identity varies from 45.26% for *Staphylococcus aureus* up to 75.15% in case of *Streptococcus pyogenes* GAPDH which implicates high variability in the protein sequence between virulent strains. In the next step we compared sequences of GAPDH peptides that were the most reactive with CDI patient sera. We found out that P9 comprise 13/16 (81.25%) unchanged amino acids in case of most homologic *Streptococcus pyogenes* and 8/16 (50%) unchanged amino acids for the most different strain which is *Staphylococcus aureus*. P10 comprises of 14/16 (87.5%) of the same amino acids in case of most homologic *Streptococcus pyogenes* and 8/16 (50%) unchanged amino acids for the most different strain which is *Staphylococcus aureus*. Surprisingly in the case of control peptide P5, which did not react with patient sera the ratio of unchanged amino acid is 10/16 (62.5%) for most similar strain which is *Streptococcus pyogenes* and 4/16 (25%) for most different strain which is *Staphylococcus aureus*. The above finding suggests that immunoreactive GAPDH peptide sequences P9 and P10 remain better preserved between strains than non-reactive peptide P5.

We confronted P9 and P10 with corresponding regions in GAPDH of strains from human gut physiologic microflora in order to verify if these peptides are widely distributed among bacterial strains. The degree of sequence preservation does not diverge from that of the pathogenic strains. The above data show that both immunoreactive sequences are very well conserved in pathogenic and non-pathogenic strains. It is easy to distinguish 100% compatible sequences such as TGAAKAI (P9) that occurs in all tested strains, regardless of homology, or GKLxGxAxRVP (P10), which are long enough to be epitopes. The control peptide P5 is characterized by much greater variability.

We compared CD GAPDH with similar protein of human origin and from mouse which is the most utilized animal model (Fig. 5). Again, we can distinguish a 6-mer identical sequence (TGAAKA) in P9 overlapped by amino acids belonging to the same subgroup (Thr-Ser are both polar uncharged amino acids; Ile and Val are both hydrophobic) which increases the length of potential epitope to 8 amino acids. We identified in P10 a conservative region composed of 11 amino acids with two 3-mers identical to human sequences (GKL and RVP). Three amino acid sequences are most probably too short to be epitopes since shortest known epitopes are 5–6 amino acid long. However, three amino acids can work as a core for antibody binding or a ‘functional epitope’ with other amino acids supporting the binding^23^.

### Peptides 9 and 10 show cross-reactivity

**Figure 6:**
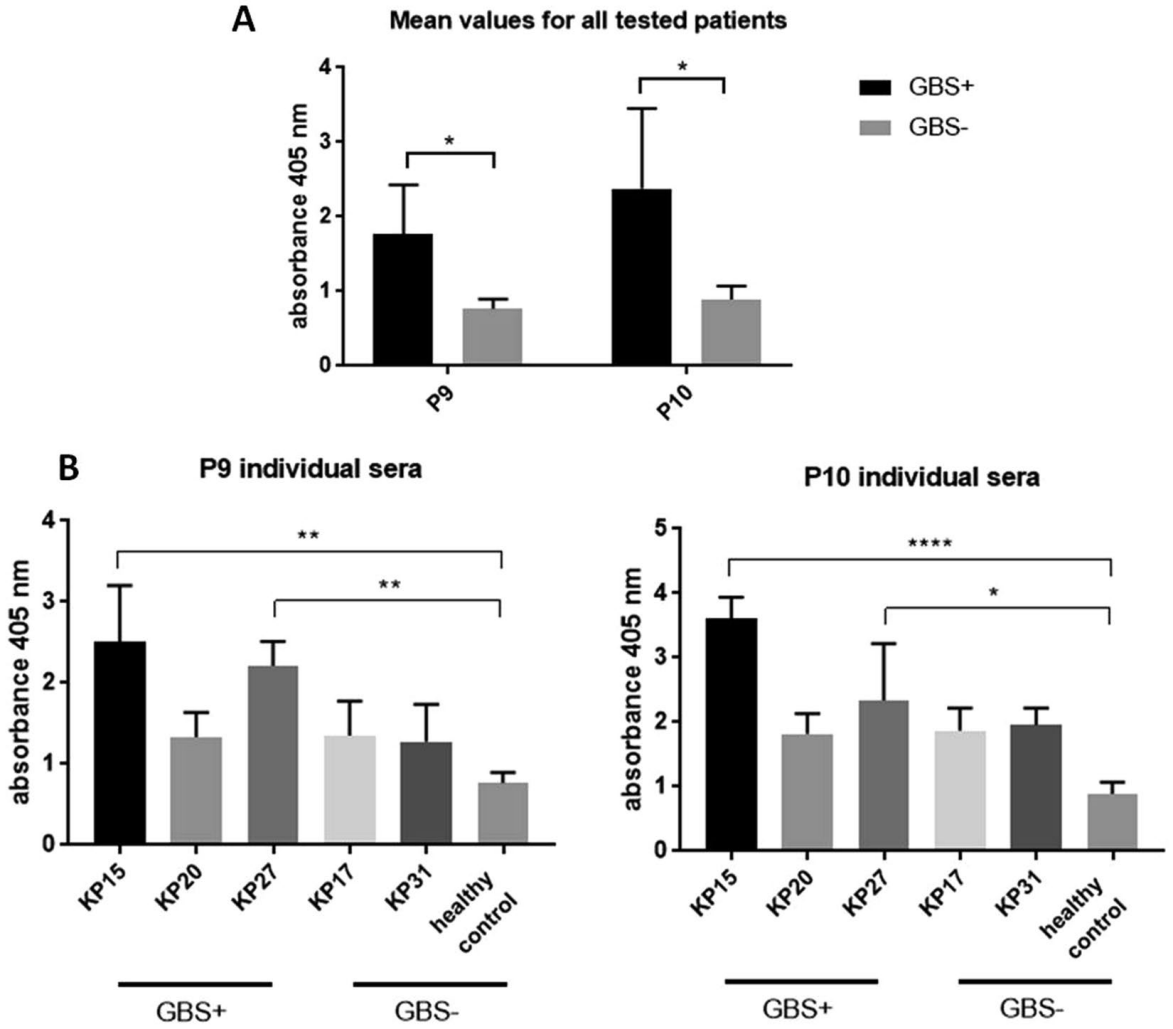
Reactivity of GBS positive umbilical cord blood sera with *C. difficile* peptides P9 and P10. (**A**) shows mean value obtained for sera GBS+ and GBS− for both peptides. (**B**) shows data for individual patients GBS+ and GBS− (healthy controls and women with other diseases) obtained for peptide P9. (**C**) shows data for individual patients GBS+ and GBS− (healthy controls and women with other diseases) obtained for peptide P10. All figures show data from at least three independent measurements, means with SD, data analyzed with 2way ANOVA *P < 0.05; **P < 0.01; ***P < 0.001; ****P < 0.0001.

In order to analyze cross-reactivity of P9 and P10 with sera immunized with other pathogens we performed ELISA using umbilical cord blood sera samples from patients diagnosed with group B streptococcus infection (*Streptococcus agalactiae*). Pooled umbilical cord blood sera samples from healthy volunteers (not diagnosed with GBS) were used as controls (healthy controls, n = 10). Experiment shows that reactivity of both peptides is significantly higher for GBS+ patients in comparison to healthy controls (GBS−) (Fig. 6A).

The analysis performed for individual patient sera shows that reactivity of P9 is significantly higher for 2 out of 3 of GBS+ patients in comparison to healthy control. Sera KP17 and KP31 were obtained from GBS− patients but with diagnosed accompanying diseases (KP17 – diabetes and Hashimoto disease, KP31 - von Willebrand disease), both show higher reactivity albeit not statistically significant when 2way ANOVA was used (Fig. 6B). The same outcome was observed for P10 analyzed with GBS+ and GBS− sera but with higher overall reactivity (Fig. 6C).

### Human sera IgG antibodies from autoimmune patients recognize epitopes of P9 and P10

What we are particularly interested in is the high degree of conservation of P9 and P10 sequences in very distant phylogenetic species such as CD and human or mouse. Despite of low homology between CD GAPDH and human GAPDH (44.5%) 10 out of 16 amino acids in sequence P9 are preserved and 8 out of 16 for P10 sequence, while with control peptide P5 only 6 out of 16 (Fig. 5). For this reason, we have decided to examine the possible relationship between these sequences and autoimmunity. We performed search in Immune Epitope Database and Analysis Resource (IEDB) in order to verify if P9 and P10 contain known epitopes. We were looking for epitope content with no less than 70% identity. The search was not restricted to any host, assay, MHC type nor disease. Results showed that fragments of P9 sequence can be found in 32 known autoepitopes, in 9 different antigens, from 5 different hosts. They were found mostly in human and mouse GAPDH. Anti-GAPDH autoantibodies specific for sequences included in peptide 9 were reported in systemic lupus erythematosus^15^ and were found in cerebrospinal fluid of patients with multiple sclerosis^14^. Fragments of P10 can be found in nucleoprotein of influenza A virus, but so far there are no known-autoepitopes in P10 sequence or verified autoantibodies.

**Figure 7:**
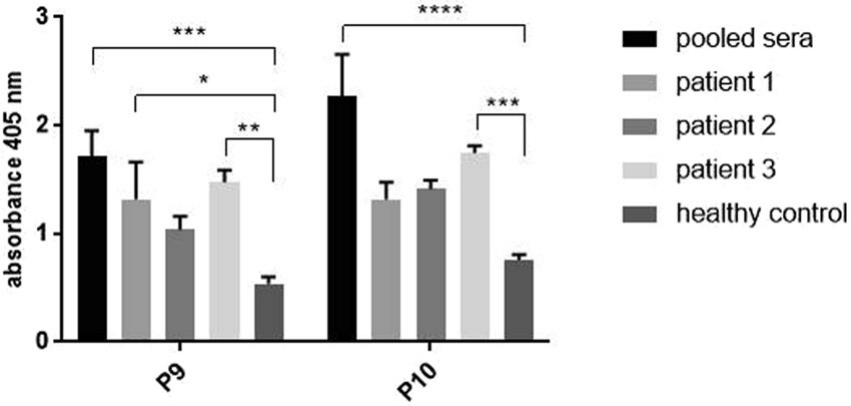
Reactivity of sera from patients with autoimmune disease with *C. difficile* peptides P9 and P10. Figure shows results from at least three independent measurements, means with SD, data was analyzed using 2way ANOVA *P < 0.05; **P < 0.01; ***P < 0.001; ****P < 0.0001.

We performed autoreactivity analysis of P9 and P10 using sera from patients with autoimmune disorder which is Graves disease (Fig. 7), an autoimmune disorder with combined still not fixed genetic/environmental origin^24^. Analysis of P9 and P10 peptides with sera from Graves’ patients (pooled, n = 10) shows elevated reactivity for both peptides in comparison with sera from healthy volunteers (pooled, n = 21). Also individual patient serum samples were analyzed. In case of P9 sera from two out of three patients show statistically significant difference in reactivity in comparison to healthy control. In case of P10 serum from one out of three patients show statistically significant difference in reactivity in comparison to healthy control. The rest of individual patient samples show higher reactivity with both peptides in comparison to healthy controls but it is not statistically significant when 2way ANOVA test is used. These results show that sera from autoimmunized patients specifically recognize both CD GAPDH peptides.

## Discussion

Finding new ways to fight *Clostridium difficile* (CD) is essential because of the increasing strains virulence and resistance to routine treatment. One way is to design an effective vaccine. At the present, there are several anti-CD vaccines in development based on toxins A and B^25^. There is also urgent need for new antigens for vaccine, preferably present on the cell surface, which would allow to wipe out the pathogen at the early stage of the disease, that is, at the time of colonization, before the pathogen even begins to produce toxins. The best solution would be to use an immunoreactive multifunctional protein critical for the bacteria survival. By using this strategy it might be possible to prevent decoy effect and protein mutation which are used by bacteria as a strategy of escaping immune response.

The search for CD new vaccine antigens was major purpose of these studies. It started with isolation of surface proteins by gentle ‘washing off’ from bacterial cell surface without its damaging. The isolate consists primarily of surface proteins^19^. Clinical strains used for the purpose of protein isolation were collected from patients to take into account currently occurring ribotypes. Western blotting analysis was performed using umbilical cord blood sera from patients without the symptoms of CDI. The use of umbilical blood sera to identify new cross-reactive proteins with other pathogens have been practiced in our Laboratory for years^20,26^. As the transmission of antibodies from mother to the baby is highly regulated and selective process^27^, and therefore the transferred antibodies have extraordinary protective potential. Results of our own study conducted on umbilical cord blood and peripheral blood sera have shown differences in profiles of recognized proteins^26^. For example, it has been shown that a natural and effective factor in protecting infants from infection is provided by IgG class antibodies, directed against GBS (group B streptococci) surface antigens, transferred from mother’s blood to the placenta^28^. Maternal IgG antibodies are partially or fully protective against respiratory syncytial virus and influenza virus in humans^29,30^. A vaccine consisting of epitopes recognized by antibodies passed from the mother could be highly protective. This is an alternative that can bring more benefits than using sera samples only from patients during active illness. Using this method we identified a set of immunoreactive proteins. One of them was assigned as type I glyceraldehyde 3-phosphate dehydrogenase (GAPDH). GAPDH was already proposed as a suitable vaccine antigen for other pathogens^9,10^ so we decided to analyze in detail the possibility of its application in vaccine.

GAPDH was considered to be a housekeeping protein of intracellular localization with metabolic functions essential for almost all organisms. Recent reports show that it might serve as a moonlight protein in various bacterial species (Supplementary Table S1) which means it is multifunctional and can exhibit alternative cellular localization. Most important from the virulence point of view is that GAPDH can bind plasminogen, mucin and fibrinogen by which can act as an adhesion molecule. We performed analysis of CD GAPDH protein sequence, its biochemical properties as well as *in silico* modeling (Fig. 2). As a result of our study we suggest that CD GAPDH is a moonlighting protein since the protein has been isolated with LiCl surface protein isolation method, has features typical for moonlighting proteins (similar sequence length, GRAVY score, theoretical pI), and displays high homology with such proteins isolated from other strains gathered in Table S1. Although, this suggestion should be supported by additional experiments. Many moonlighting proteins fulfill additional functions, very often are directly associated with virulence and can be located on the surface of the cell where the therapeutic antibodies are readily accessible which justify the usage of such proteins as an vaccine antigens. Moonlighting properties of CD GAPDH should be further investigated since it might be important for CD virulence.

Epitope mapping of CD GAPDH using sera from patients with CDI revealed two highly immunoreactive peptides P9 and P10, which were further investigated for cross-reactivity and autoimmunoreactivity. Patient sera were used instead of umbilical cord blood sera for epitope mapping in order to check the response of strongly immunized patients. The key findings of this publication are that both peptides are common and conserved between pathogenic and non-pathogenic strains (Fig. 5); both epitopes are specifically recognized by sera from GBS+ patients (Fig. 6) and patients with autoimmune disease (Fig. 7) which excludes their usage as vaccine antigens.

A potential vaccine epitope should be specific for the pathogenic strain and not to non-pathogenic strains to avoid cross-reactivity, especially with bacteria naturally present in the body. The level of sequence similarity of both peptides was investigated among pathogenic strains expressing the protein as a moonlight protein, strains most prevalent in gut flora, with human and mouse GAPDH sequences (Fig. 5). P9 and P10 show high level of similarity between pathogenic and healthy gut flora. This may indicate that this part of the protein is responsible for the function of the protein and therefore stays conservative. So far there is only one well known active site in GAPDH which is localized around Cys_149_ and His_176_^31^ and falls far from the localization of P9 and P10. These sequences may have another functionalities for example maintain protein structure or take part in interactions with other proteins. GAPDH as a moonlighting protein comes into interaction with other proteins both intracellular and extracellular, including plasminogen and mucin^32–34^. Moreover, we still do not know how the GAPDH is maintained on the surface of the bacterial cell where it fulfills the above-mentioned adhesion functions. The contribution of such interactions of both peptides should be investigated.

We found out that umbilical cord blood sera isolated from patients with GBS infection recognize specifically P9 and P10 (Fig. 6). GBS GAPDH is already well described in the literature^33,35^. In this pathogen GAPDH is an extracellular virulence factor that induces IL-10 expression which has immunosuppressive effect on the host by impairing neutrophil recruitment to infected sites^36^. Maternal vaccination with GBS GAPDH protects newborns against infection. Antibodies against P9 and P10 might have protective properties which should be further investigated. However, before administration of such a vaccine to a human subject the possible autoimmunizing properties should be taken into account.

The GAPDH-connected situation gets even more complicated when probiotic bacteria are added into the equation. The protein is not only found on the surface of microbiome bacteria but also shows immunoreactivity which was reported in our previous studies^37^. We identified GAPDH among many other immunoreactive moonlighting proteins in *Bifidobacterium longum* ssp. *longum* CCDM 372. The same protein was found on the surface of numerous lactic acid probiotic bacteria: *L. acidophilus*, *Lactobacillus amylovorus*, *L. crispatus*, *Lactobacillus gallinarum*, *L. gasseri*, *L. johnsonii*, *Lactobacillus paracasei*, *L. rhamnosus* and *L. lactis* in which it was associated with pH-dependent adhesion to plasminogen and mucin. Our preliminary data show that sera from rabbits immunized with *Lactobacillus johnsonii* 142^38^ does not specifically recognize P10 and P9 but other peptides from GAPDH sequence exhibit higher immunoreactivity (data not shown). It might be explained by that immune systems from different organisms elicit alternative antibody population to the same antigen^20^ or that probiotic strains modulate immune response in such way that antibodies of different specificity are produced.

What we are particularly interested in is the high degree of conservation of P9 and P10 sequences in very distant phylogenetic species such as CD and human or mouse. Despite GAPDH whole protein homology about 44.5% for human 10 out of 16 amino acids in sequence P9 are preserved and 8 out of 16 amino acids in P10 sequence, with control peptide P5 only 6 amino acids out of 16 (Fig. 5). For this reason, we have examined the possible relationship between these sequences and autoimmunity which revealed anti-GAPDH autoantibodies specific for sequences existing in peptide 9. These sequences were reported in systemic lupus erythematosus^15^ and were found in cerebrospinal fluid of patients with multiple sclerosis^14^. We have shown that pooled sera collected from patients with autoimmune disease (Graves disease, n = 10) are more reactive with both peptides tested as compared to healthy controls (n = 10). Serum samples from two out of three individual patients showed greater immunoreactivity with P9 than healthy controls.

Peptides from microorganisms, that are sufficiently similar to the host antigens, can activate autoreactive T cells. This phenomenon is called molecular mimicry^39,40^. In particular, inflammation and recurrence of infection, which is very common in CDI, can lead to localized activation of antigen presenting cells and to increased processing and presentation of own antigens present in the site of inflammation. An example of an autoimmune disease caused by acute infection is Guillain-Barré syndrome, a disease that attacks various organs including the central nervous system. The etiological factor of the disease is *Campylobacter jejuni*, the leading cause of diarrhea in the United States. *C. jejuni* induces antibodies that cross-react with peripheral nerve antigens^41^. We find an analogy to CDI in the above-mentioned phenomenon. Strong infection such as CDI may have more effect on the host immune system than we thought. Probably the immune system mobilizes all the strengths to clear off the infection but at the same time it can lead to subsequent serious complications. Antibodies that target host proteins or natural bacterial flora may appear during strong inflammation caused by CD, which later result in the appearance of an autoimmune disease. It seems that other pathogens may affect the system in similar way since we demonstrated a cross-reaction between P9 and P10 with sera collected from GBS+ patients, but in this case we have not done limited dilution of antibody titer. A massive infection like CDI elicits a burst of antibodies which cannot be compared with the yield of umbilical cord transport or the level of antibodies induced by gut flora in which case it can have protective or desensitizing role.

Autoimmune response as a result of vaccination albeit very rare should be taken into consideration when developing a new vaccine. Several cases described the relationship between vaccination against hepatitis B and demyelization, which can lead to multiple sclerosis^42,43^. There have also been isolated cases linking hepatitis B vaccine and rheumatoid arthritis^44^. Another study show that after infection with *Borrelia burgdorferi*, some vaccines are more likely to develop chronic, poorly responsive Lyme arthritis associated with high levels of antibody to the vaccine antigen OspA, in serum and synovial fluid^45^.

Finding suitable bacterial vaccine antigen is a complicated process due to multiple mechanisms used by pathogens to avoid immune system and because of the possibility of eliciting an immune response against own antigens or antigens of own bacterial flora. One of the recently proposed protein is GAPDH which is essential and conserved. Above experiments show how to verify the suitability of a protein as a vaccine antigen using GAPDH from *Clostridium difficile* as an example. We demonstrated that infection with this pathogen may cause humoral response to produce anti-GAPDH antibodies which are cross-reactive with GAPDH sequences of other pathogens and/or healthy gut microflora. Furthermore, CD GAPDH specific epitopes may induce antibodies directly against self-antigens leading to autoimmunity which is why it should not be used in a vaccine as a whole protein. However, it does not preclude the possibility that GAPDH yet contains other epitopes that might be utilized as antigen to produce robust and protective vaccine but further investigation is warranted. As a result of the analysis we suggest that using GAPDH as a vaccine antigen should be thoroughly investigated in the case of each pathogen.

## Materials and Methods

### Blood sera

#### Human peripheral blood sera

Blood sera (pooled n = 15) from patients diagnosed with CDI (based on ≥3 loose stools in 24 hours, abdominal pain, fever and positive laboratory results for presence of GDH - glutamate dehydrogenase and CD toxins A and B (C. Diff Quik Chek Complete; TECHLAB, Inc, USA)^46^) were obtained from 4^th^ Military Hospital in Wroclaw, written approval was received from the Medical Ethics Commission of the Medical University of Wroclaw (no. KB-631/2015) and were conducted in accordance with the Helsinki Declaration, 1975. Samples were obtained with patients who signed the written informed consent.

Blood sera from patients with autoimmune disorders (n = 10) were obtained from unrelated patients sufferring from Graves’ disease (GD). Samples were obtained with patients who signed the written informed consent. The initial diagnosis of GD was confirmed by routine clinical and laboratory tests, including a history of thyrotoxicosis, diffuse goiter, suppressed serum TSH, elevated free thyroxine, and/or free triiodothyronine levels, as well as high anti-thyroid peroxidase or TSH-binding inhibiting immunoglobulin titers. The ophthalmological examination, together with the severity and ocular activity, was performed at the time of blood collection^47^. Three individual patient serum samples obtained from not smoking females with sporadic incidence of GD and Graves’ orbitopathy (GO) were selected for separate analysis. Two of them presented active GO, and one - severe GO at the day of blood sampling. Sera used as healthy controls (pooled, n = 21) were collected from apparently healthy volunteers. Performed experiments were approved by the Medical Ethics Committee of the Medical University of Wroclaw (approval No KB-322/2010) with patients’ and healthy volunteers written informed consent and were conducted in accordance with the Helsinki Declaration, 1975.

#### Human umbilical cord blood sera

Human umbilical cord blood sera (pooled n = 10) used for immunoreactive protein screening were obtained from the Obstetric Clinic of the Medical University of Wroclaw (approval no. KB-882-2012), collected from healthy women. Samples were obtained with patients who signed the written informed consent and were conducted in accordance with the Helsinki Declaration, 1975. The same sera were used as healthy controls for GBS experiment.

Umbilical cord blood sera for cross-reactivity testing with GBS were obtained from pregnant women with confirmed genital tract and/or anus colonization by *Streptococcus agalactiae*. The inclusion criteria were: pregnant women in the third trimester between 18 and 40 years of age; a written statement of consent to participate in the study. Sera characterization: KP15 GBS+, Hashimoto disease; KP20 GBS+, hypothyroidism; KP 27 GBS+, diabetes; KP17 GBS−, Hashimoto disease, diabetes; KP31 GBS−, Von Willebrand disease. The study was approved by Jagiellonian University Bioethical Committee decision no. KBET/153/B/2014. Samples were obtained with patients who signed the written informed consent and were conducted in accordance with the Helsinki Declaration, 1975.

### Bacterial strains and culturing conditions

*Clostridium difficile* Cd20 strain was isolated from a stool sample of a 71 year old patient diagnosed with pseudomembranous colitis, according to clinical symptoms, laboratory (as described above) and colonoscopy results. Stool sample was cultured anaerobically at least for 48 h on selective CD media [CDIF - chromID™ *C. difficile* and CLO - Columbia agar with cycloserine, cefoxitin and amphotericin B (bioMerieux, Mary L’Etoile, France)] incubated for at least 48 hours in a temperature of 37 °C under anaerobic conditions in the anaerobic chamber. Colonies were examined for the characteristic horse odor, yellow-green fluorescence in the UV light and Gram staining. Suspected colonies were identified by the ANC card in an automatic system - VITEK 2 Compact. Toxin production (toxins TcdA, TcdB and binary toxins) was determined by molecular methods, and ribotyping was performed, as previously described^46,48^. CD strain belongs to ribotype 027, and was resistant to moxifloxacin, ciprofloxacin, erythromycin and imipenem, as was demonstrated by E-tests^46^. Strain was deposited in Polish Collection of Microorganisms as PCM 2826. The isolate was cultured in BHI with addition of 0.05% L-cysteine hydrochloride at 37 °C in anaerobic conditions for 48 h. Each time culture was inspected in terms of microbiological content. Cells were harvested by centrifugation in (6000 × g for 15 min) and washed two times in phosphate-buffered saline pH 7,4 (PBS) before used in further experiments.

### Surface proteins isolation

Surface proteins of CD were isolated using 1 M LiCl method which offers surface protein isolation in mild conditions^19^. 1 g of PBS-washed bacterial mass was suspended in 7 ml of freshly prepared 1 M LiCl. The mixture was incubated for 1 h in room temperature with shaking. After centrifugation (6000 × g for 5 min) supernatant was collected and dialyzed against MQ water for at least 48 h and concentrated on filters MWCO = 10 000 Da. The concentration of surface proteins was estimated using Pierce™ BCA Protein Assay Kit (Thermo Fisher Scientific). Prepared samples were aliquoted and stored at −20 °C.

### SDS-PAGE and Western blotting

SDS-PAGE was performed according to Laemmeli^49^ using large size gels 18 × 16 cm with 1.5 mm thick combs, resolved in SE600 Standard Dual Cooled Vertical Unit. Each time equal amount of proteins was loaded on the 5–12.5% gels (30 µg per well) according to Laemmli^49^, Tris-Glycine-SDS was used as running buffer. Each time a double-stained Precision Plus Protein Dual Color protein ladder was loaded on the gel (Bio-Rad). Gels were run for about 5 hours at 150 V with cooling. Protein samples for silver staining and Western blotting were run on one gel which was cut into two parts after electrophoresis. Silver staining was performed according to Shevchenko with changes^50^. Gel was fixed in 5% acetic acid and 50% methanol for 60 min, washed in 50% methanol and MQ water, incubated 3 min in 0.02% sodium thiosulfate, washed again with MQ water, incubated for 45 min in 0.15% silver nitrate washed with MQ water, the reaction was induced by 2% sodium carbonate, and 0.04% formaldehyde. The reaction was stopped by 5% acetic acid. The second part of the gel was incubated in transfer buffer (10 mM Tris-HCl, 150 mM glycine, 20% methanol, pH 8.3) for 30 min and transferred into polyvinylidene difluoride membrane Immobilon-P using Trans-Blot® SD Semi-Dry Transfer Cell. Transfer condition was 25 V for 60 min. The efficiency of transfer was tested by silver staining the remaining gel. Western blotting was done as previously^37^. Membrane with transferred proteins was blocked in 1% bovine serum albumin in Tris-buffered saline with Tween 20 (TBST, 20 mM Tris, pH 7.5, 150 mM NaCl, 0.1% Tween 20) for 1 h in RT. We used umbilical cord blood sera for immunoblotting, dilution 1:100 in TBST with 0.1% BSA, incubated overnight at 4 °C. Membrane was washed three times in TBST 10’ with mixing and incubated with secondary antibodies, anti-human IgG conjugated with AP diluted 1:10 000, 1 h in RT. Membrane was washed three times in TBST 10’ with mixing and reaction was induced by adding solution containing nitro blue tetrazolium and 5-bromo-4-chloro-3-indolyl phosphate in TBST with MgCl_2_. Reaction was developed until bands become visible and then stopped by washing in MQ water. Membrane was documented using PXi imaging system using protocol for visible blots.

### Protein identification

Protein bands cut from the gel were identified using protein digestion with trypsin followed by separation of peptides mixture by liquid chromatography and mass spectrometry of the peptides and peptide fragments by the mass spectrometer LC-MS-MS/MS Orbitrap. Peptide molecular masses were compared with the protein sequence database (NCBI, UniProt database) using MASCOT program (http://www.matrixscience.com/). All searches were performed against the database for *Peptoclostridium difficile*.

### Bioinformatic analysis

#### GAPDH protein structure modeling

Since there is no crystal structure for GAPDH from CD the structure model was built using homological sequence. The sequence of the highest homology (74.55% identity) with known crystal structure was found by BLAST - *Streptococcus agalactiae* GBS GAPDH (PDB 5jyf.1.A, crystal structure obtained using x-ray diffraction at 2.62 Angstrom resolution^51^). The model was built by SWISS MODEL workspace^52^ and used for selecting potential epitopes.

#### GAPDH amino acid sequence analysis

The sequence variation between 50 different CD strains, including toxigenic and nontoxigenic, was performed using Clustal Omega^53^. The same tool was used to align CD GAPDH sequence with sequences of the same protein from other pathogenic and nonpathogenic strains. Subcellular localization was predicted using PSORTb v3.0 (Gram-positive bacteria)^54^. It employs SCL predictors using 5-fold cross validation and performs proteomics analysis which results in most precise localization predictor.

#### GAPDH epitope prediction

Linear B-cell epitopes were predicted using two online tools BCPred and SVMTrip. Each time linear 16-aa epitopes were predicted. BCPred predict linear B-cell epitopes using Support Vector Machine classifiers that use string kernels^55^. SVMTrip is a method in which Support Vector Machine (SVM) has been utilized by combining the Tri-peptide similarity and Propensity scores (SVMTriP) in order to achieve the better prediction performance^56^. T lymphocyte epitope mapping tools were also used to designate potential epitopes (http://crdd.osdd.net/raghava/mhc/index.html).

Results from structure analysis and epitope prediction tools were combined into twenty 16-mer amino acid epitopes selected for chemical synthesis on plastic pins.

#### Immune epitope database and autoantigen search

Immune Epitope Database and Analysis Resource (IEDB, www.iedb.org)^57^ was used for searching for known epitopes within analyzed peptides. We were looking for epitope content with no less than 70% identity. The search was not restricted to any host, assay, MHC type nor disease. AAgAtlas database 1.0 was used for autoantigen search (http://biokb.ncpsb.org/aagatlas). This database was used for browsing of autoantigens and associated diseases.

### Epitope mapping

#### Epitope synthesis

Selected peptides were synthesized using Geysen (PEPSCAN) procedure with changes^20,58^. Peptides were synthesized on polyethylene pins (MIMOTOPES, Clayton, Victoria, Australia) in a 96 well plate by adding one amino acid to each pin during one coupling reaction. F-moc amino acid derivatives with blocked side groups were used for the synthesis. Pins underwent deprotection in 20% piperidine in dimethylformamide (DMF) before the addition of each amino acid. Then, after washing in DMF and methanol dried pins were subjected to the coupling reaction. The reaction mixture consisted of 60 mM of blocked amino acid, 60 mM diisopropylcarbodiimide and 65 mM N-hydroxybenzotriazole in DMF. 0.5 mM bromophenol blue were added to monitor the progress of the reaction. The coupling reaction was carried out for at least 4 hours at room temperature in a sealed vessel. After washing and another pin deprotection it was subjected to the next coupling reaction until a full-length peptide was obtained. The full length pin-bound peptide was subjected to the side group’s removal reaction with 2.5% anisole and 2.5% dithioethane in trifluoroacetic acid. Peptides were then washed in methanol, 0.5% acetic acid and methanol again, dried and stored at −20° until used.

#### Enzyme-Linked Immunosorbent Assay (ELISA)

Epitopes were mapped by ELISA performed on pin-bound peptides using sera from different groups as previously^20^. Before the assay, pins were equilibrated in TBS-T for 10 min. Pins were blocked for 1 h in RT with 1% bovine serum albumin. Sera were used in 1:1000 dilution in TBS-T, secondary antibodies anti-human IgG conjugated with AP were used in 1:10000 dilution (Sigma-Aldrich). Color reaction was performed by using Alkaline Phosphatase Yellow Liquid Substrate System for ELISA. All assays were performed at least in triplicate. Bound antibodies were detached by 10 min sonication in disruption buffer, pH 7.2 heated to 60 °C consisting of 1% sodium dodecyl sulfate, 0.1% 2-mercaptoethanol and 0.1 M Na_3_PO_4_. After washing in water and methanol pins were dried and stored at 4 °C until further used.

### Statistical analysis

All figures show data from at least three independent measurements, means with SD, data analyzed with 2way ANOVA. Statistical analysis was performed using GraphPad Prism version 7.

## Electronic supplementary material

Supplementary information

## Data Availability

The datasets generated during and/or analyzed during the current study are available from the corresponding author on reasonable request.
