# Supplementary material for: Epitopes identified in GAPDH from Clostridium difficile recognized as common antigens with potential autoimmunizing properties
Source: Sci Rep. 2018 Sep 17;8:13946. doi: 10.1038/s41598-018-32193-9 (PMC6141484; doi:10.1038/s41598-018-32193-9)
Supplement: Supplementary file 1 — Supplementary information [file 41598_2018_32193_MOESM1_ESM.pdf]

Supplementary information

Epitopes identified in GAPDH from *Clostridium difficile* recognized as common antigens with potential autoimmunizing properties

Razim A.*[1], Pacyga K.[1], Aptekorz M.[2], Martirosian G.[2], Szuba A.[3,4], Pawlak-Adamska E.[5], Brzychczy-Włoch M.[6], Myc A.[1,7], Gamian A.[1], Górska S.[1]

[1] Hirszfeld Institute of Immunology and Experimental Therapy, Polish Academy of Sciences, Department of Immunology of Infectious Diseases, Laboratory of Medical Microbiology, Wroclaw, Poland

[2] Department of Medical Microbiology, School of Medicine in Katowice, Medical University of Silesia, Katowice, Poland

[3] Division of Angiology, Wroclaw Medical University, Wroclaw, Poland

[4] Department of Internal Medicine, 4th Military Hospital in Wroclaw, Poland

[5] Hirszfeld Institute of Immunology and Experimental Therapy of the Polish Academy of Sciences, Department of Experimental Therapy, Laboratory of Immunopathology, Wroclaw, Poland

[6] Department of Molecular Medical Microbiology, Chair of Microbiology, Jagiellonian University Medical College, Krakow, Poland

[7]Research Associate Scientist Emeritus, University of Michigan, Nanotechnology Institute for Medicine and Biological Sciences, Ann Arbor, MI 48109, U.S.A.

Contact: agnieszka.mehlich@iitd.pan.wroc.pl

Figure S1

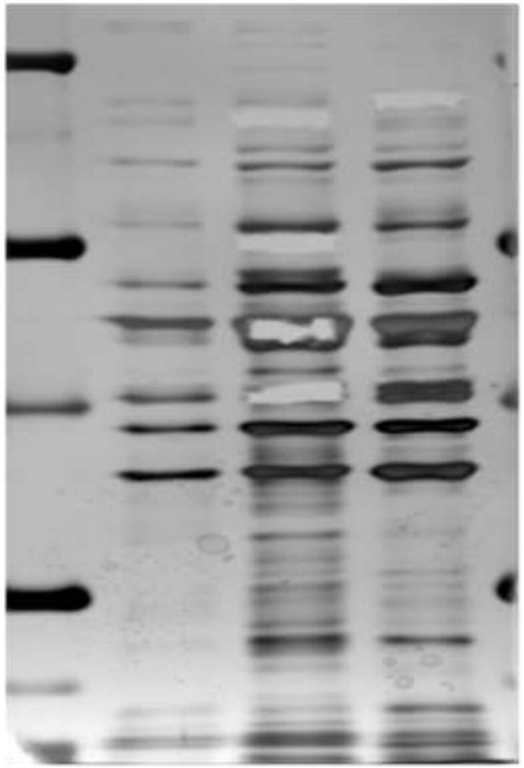

Figure S1 Uncropped gel used in Figure 1

Figure S2

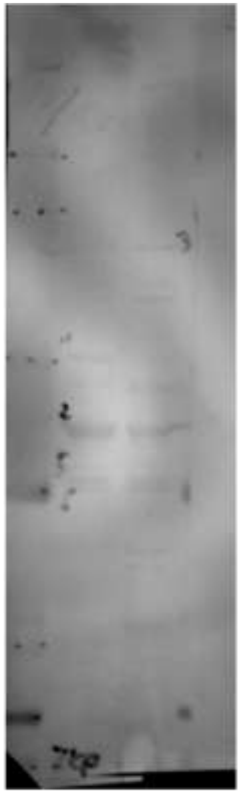

Fig S2 Picture of the original blot used in Figure 1

Table S1 GAPDH as moonlighting protein.

organism	alternative function	reference
Escherichia coli	NAD ribosylating activity	(Aguilera i in. 2009)
Lactobacillus plantarum	binds mucin and Caco-2 cells	(Kinoshita i in. 2008)
Lactococcus lactis IL1403	binding to invertase, a hyperglycosylated mannoprotein from Saccharomyces cerevisiae	(Katakura i in. 2010)
Streptococcus anginosus and oralis	plasminogen binding	(Kinnby, Booth, i Svensäter 2008)
Streptococcus pyogenes	binds uPAR/CD87 receptor on human cells	(Jin i in. 2005)
Streptococcus suis (serotype 2)	plasminogen binding	(Jobin i in. 2004)
Trichomonas vaginalis	Fibronectin, plasminogen, collagen binding	(Lama i in. 2009)
Staphylococcus epidermidis and aureus	transferrin-binding protein (also called TPN) and also binds plasminogen involved in the acquisition of transferrin-bound iron	(Modun i Williams 1999)
Streptococcus pneumoniae	plasminogen binding	(Bergmann, Rohde, i Hammerschmidt 2004)
Streptococcus agalactiae, group B, serotype III/deltaepsilon, strain J48	plasminogen binding	(Seifert i in. 2003)
Bacillus anthracis	plasminogen binding	(Matta, Agarwal, i Bhatnagar 2010)
Mycoplasma genitalium	binds mucin	(Alvarez, Blaylock, i Baseman 2003)

Aguilera, L., Giménez, R., Badia, J., Aguilar, J. & Baldoma, L. NAD+-dependent post-translational modification of Escherichia coli glyceraldehyde-3-phosphate dehydrogenase. *Int. Microbiol. Off. J. Span. Soc. Microbiol.* **12,** 187–192 (2009).

Alvarez, R. A., Blaylock, M. W. & Baseman, J. B. Surface localized glyceraldehyde-3-phosphate dehydrogenase of Mycoplasma genitalium binds mucin. *Mol. Microbiol.* **48,** 1417–1425 (2003).

Bergmann, S., Rohde, M. & Hammerschmidt, S. Glyceraldehyde-3-Phosphate Dehydrogenase of Streptococcus pneumoniae Is a Surface-Displayed Plasminogen-Binding Protein. *Infect. Immun.* **72,** 2416–2419 (2004).

Jin, H., Song, Y. P., Boel, G., Kochar, J. & Pancholi, V. Group A Streptococcal Surface GAPDH, SDH, Recognizes uPAR/CD87 as its Receptor on the Human Pharyngeal Cell and Mediates Bacterial Adherence to Host Cells. *J. Mol. Biol.* **350,** 27–41 (2005).

Jobin, M.-C., Brassard, J., Quessy, S., Gottschalk, M. & Grenier, D. Acquisition of Host Plasmin Activity by the Swine Pathogen Streptococcus suis Serotype 2. *Infect. Immun.* **72,** 606–610 (2004).

Katakura, Y., Sano, R., Hashimoto, T., Ninomiya, K. & Shioya, S. Lactic acid bacteria display on the cell surface cytosolic proteins that recognize yeast mannan. *Appl. Microbiol. Biotechnol.* **86,** 319–326 (2010).

Kinnby, B., Booth, N. A. & Svensäter, G. Plasminogen binding by oral streptococci from dental plaque and inflammatory lesions. *Microbiology* **154,** 924–931 (2008).

Kinoshita, H. *et al.* Cell surface Lactobacillus plantarum LA 318 glyceraldehyde-3-phosphate dehydrogenase (GAPDH) adheres to human colonic mucin. *J. Appl. Microbiol.* **104,** 1667–1674 (2008).

Lama, A., Kucknoor, A., Mundodi, V. & Alderete, J. F. Glyceraldehyde-3-Phosphate Dehydrogenase Is a Surface-Associated, Fibronectin-Binding Protein of Trichomonas vaginalis. *Infect. Immun.* **77,** 2703–2711 (2009).

Matta, S. K., Agarwal, S. & Bhatnagar, R. Surface localized and extracellular Glyceraldehyde-3-phosphate dehydrogenase of Bacillus anthracis is a plasminogen binding protein. *Biochim. Biophys. Acta BBA - Proteins Proteomics* **1804,** 2111–2120 (2010).

Modun, B. & Williams, P. The Staphylococcal Transferrin-Binding Protein Is a Cell Wall Glyceraldehyde-3-Phosphate Dehydrogenase. *Infect. Immun.* **67,** 1086–1092 (1999).

Seifert, K. N., McArthur, W. P., Bleiweis, A. S. & Brady, L. J. Characterization of group B streptococcal glyceraldehyde-3-phosphate dehydrogenase: surface localization, enzymatic activity, and protein–protein interactions. *Can. J. Microbiol.* **49,** 350–356 (2003).
